# Sex-Specific Associations Between Lifestyle Factors and Sick Leave in the Serbian Working Population: Findings from the National Health Survey

**DOI:** 10.3390/healthcare12222203

**Published:** 2024-11-05

**Authors:** Snezana Knezevic, Tamara Gajic, Nela Djonovic, Sara Knezevic, Dragan Vukolic, Tatjana Marinkovic, Nikoleta Janicijevic, Dragan Vasiljevic, Slavica Djordjevic, Dragan Marinkovic, Dalibor Stajic

**Affiliations:** 1 Academy of Applied Studies Polytechnic, 11000 Belgrade, Serbia; tmarinkovic@politehnika.edu.rs; 2 Doctoral Academic Studies-Medical Sciences, Faculty of Medical Sciences, University of Kragujevac, 34000 Kragujevac, Serbia; 3 Geographical Institute “Jovan Cvijić″, Serbian Academy of Sciences and Arts, 11000 Belgrade, Serbia; tamara.gajic.1977@gmail.com; 4 Institute of Environmental Engineering, Peoples’ Friendship University of Russia (RUDN University), 101000 Moscow, Russia; 5 Department of Hygiene and Ecology, Faculty of Medical Sciences, University of Kragujevac, 34000 Kragujevac, Serbia; nela.djonovic@gmail.com (N.D.); nikoleta.janicijevic@gmail.com (N.J.); dvg_gana@yahoo.com (D.V.); 6 Doctoral Academic Studies-Intelligent Software Engineering, Faculty of Technical Sciences, Singidunum University, 11000 Belgrade, Serbia; sara.knezevic.23@singimail.rs; 7 Faculty of Tourism and Hotel Management, University of Business Studies, 78000 Banja Luka, Bosnia and Herzegovina; vukolicd@yahoo.com; 8 Department of the High School of Health, Academy of Applied Studies Belgrade, 11000 Belgrade, Serbia; slavica.djordjevic30@gmail.com; 9 Faculty of Special Education and Rehabilitation, University of Belgrade, 11000 Belgrade, Serbia; dragan.marinkovic@hotmail.com

**Keywords:** sex differences, body mass index, smoking, alcohol consumption, exercise, health surveys, sick leave

## Abstract

**Background/Objectives**: Sick leave is influenced by various modifiable lifestyle factors and sex differences. This study investigates the associations between body mass index, fruit and vegetable consumption, physical activity, smoking, and alcohol consumption and sick leave among Serbia’s working population, with emphasis on sex differences. **Methods**: Data from the 2019 National Health Survey of Serbia were analyzed, involving a sample of 4652 individuals. Chi-square tests and logistic regression models assessed the relationships between lifestyle factors and sick leave. **Results**: Among the participants, 15.8% reported sick leave in the past 12 months, with higher rates among women in both short-term (13.9% vs. 10.6%) and long-term (3.4% vs. 2.2%) sick leave. This study identifies obesity (OR = 2.6), poor dietary habits (fruit OR = 2.1; vegetables OR = 2.8), smoking (OR = 1.9), and risky alcohol consumption (OR = 4.1) as key predictors of sick leave in men, while smoking (OR = 1.8) and risky alcohol consumption (OR = 3.1) are major predictors in women. The inconsistent association between diet, physical activity, and sick leave may be attributed to differences in reporting accuracy, differing definitions of healthy intake, or the influence of unmeasured lifestyle factors. **Conclusions**: Smoking and risky alcohol consumption increase the odds of sick leave for both sexes. Interventions targeting smoking cessation and mitigating risky alcohol consumption could significantly decrease sick leave rates. While fruit and vegetable consumption, along with physical activity, showed inconsistent effects in both sexes, further studies are warranted to elucidate their roles.

## 1. Introduction

Sick leave presents a significant challenge in occupational and public health, with various modifiable lifestyle factors playing a crucial role [1]. Lifestyle behaviors including obesity, dietary habits, low physical activity, smoking, and alcohol consumption contribute to lost work years due to disability and premature mortality [1,2,3]. These unhealthy lifestyle choices can result in chronic conditions including obesity, cardiovascular diseases, and mental health disorders, increasing the likelihood of sick leave [3]. Obesity is associated with various chronic conditions that necessitate time off work [1,3]. Poor dietary habits increase susceptibility to illness, while low physical activity is associated with obesity and other health problems [3]. Smoking contributes to chronic diseases, and excessive alcohol consumption can result in liver issues and mental health disorders [3]. Collectively, these behaviors contribute to higher absenteeism rates, accounting for 15–30% of all sick leave [2,3,4] and 20% of long-term sick leave cases [5]. The interplay of multiple health-related lifestyle factors exacerbates existing health conditions and increases the odds of both short-term and long-term sick leave, particularly through the combination of smoking, high body mass index (BMI), and risky alcohol consumption [5]. Despite well-established associations between modifiable lifestyle factors and sick leave [1,2,3,4,5], there remains a significant gap in comprehensive studies examining their combined associations across various populations and occupational settings [3]. This gap underscores the urgent need for more in-depth studies to identify at-risk groups and develop targeted interventions to reduce sick leave and enhance workforce health outcomes. Additionally, the sex disparity in sick leave, as noted by Østby et al. [6], remains an important and largely unexplained issue that warrants further investigation.

High BMI is strongly associated with increased sick leave [5,6,7,8]. Both overweight and obesity significantly increase the odds of sick leave in both sexes, with individuals having a BMI of 25 kg/m^2^ or higher showing higher rates of sick leave compared to non-obese individuals [7]. Central obesity, characterized by excess abdominal fat, further exacerbates sick leave due to associated health problems [9]. In Europe, higher BMI is particularly associated with increased sick leave in southern and western countries, including Malta, Italy, Spain, and France, while the weakest association was observed in Romania [1]. Changes in weight, including both weight gain and loss, are significant predictors of sick leave [10].

While obesity plays a significant role in sick leave, the influence of dietary factors alone is less pronounced [5]. High-quality diets, rich in fruits and vegetables, may reduce sick leave, but these associations are minimal without additional lifestyle changes [5,6]. Although some studies suggest that such diets can significantly decrease sick leave [9], the overall evidence is limited, and dietary habits generally have a minor association with sick leave [4,5]. Despite the Mediterranean diet’s known benefits for disease prevention, a Spanish study found no significant link between this diet and reduced sick leave [11]. Poor dietary habits may increase sick leave, particularly when combined with low physical activity [5].

Low physical activity is strongly associated with increased sick leave [11,12,13,14]. The relationship between physical activity and sick leave is complex, demonstrating both positive and negative associations [9,12,15]. For example, a Spanish study found that high levels of leisure-time physical activity reduced sick leave, especially among women [16]. However, the findings vary due to differences in outcome measures, job types, and working conditions [10,17]. Intensive physical activity is associated with significantly shorter sick leave compared to individuals who are sedentary or lightly active [11].

Tobacco consumption is a significant lifestyle factor associated with increased sick leave [14,17]. Daily smokers have 30% higher odds of experiencing frequent and prolonged sick leave compared to non-smokers [17]. This strong association persists across various demographics and study conditions, although some studies report inconsistent results [12,15,18]. The association is especially pronounced in men, likely due to higher smoking rates [17]. Despite some variability, daily smoking consistently correlates with higher rates of sick leave [19].

Alcohol consumption is a significant lifestyle factor influencing sick leave [14,17]. The relationship between alcohol consumption and sick leave varies by outcome and sex. High alcohol consumption is sometimes associated with fewer sick leave days compared to non-drinkers [17], but this association is more pronounced for long-term sick leave among men [20]. However, the findings are inconsistent, with some studies showing no significant link between alcohol consumption and sick leave [11,17]. Overall, the association between alcohol consumption with sick leave is complex and varies, particularly influencing long-term sick leave in men [20].

Unhealthy lifestyle behaviors vary across occupational and socioeconomic groups, with skilled manual workers, self-employed individuals, and individuals working long hours exhibiting higher odds of sick leave [21,22]. These groups are more likely to smoke, consume alcohol heavily, and be sedentary, contributing to increased sick leave rates [20]. While office workers tend to be more inactive and drink heavily, they smoke less [21]. Agricultural workers generally have decreased odds of these behaviors, whereas shift workers are more prone to smoking [21]. Some studies find no significant differences in lifestyle behaviors across occupational groups, underscoring the need for more nuanced studies [21].

Sex differences in sick leave are notable, with women generally exhibiting higher absenteeism rates than men [1,23]. Lifestyle factors including smoking, obesity, and alcohol consumption demonstrate varying associations with sick leave by sex [24]. Overweight and obese women tend to have higher sick leave rates compared to men [7,25,26]. However, some studies suggest that sex differences in the association between these lifestyle factors and sick leave may be less pronounced [5,24]. Alcohol consumption particularly influences long-term sick leave in men, contributing significantly to the disparities in sick leave rates between manual and non-manual workers [20].

Exposure to multiple health-related risk factors including low physical activity, poor dietary habits, obesity, alcohol consumption, and smoking is strongly associated with higher sick leave rates [4,5,25]. Improving these lifestyle factors according to health recommendations could enhance workability and reduce sick leave-associated costs [3,4,26]. Previous research indicates that workplace interventions targeting nutrition and physical activity can reduce sick leave and improve work capacity [27]. However, evidence on how these factors interact with sick leave remains inconsistent. Some studies have found no association between obesity and sick leave across both sexes [15] or specifically among men [28]. Additionally, another study shows no significant link between physical activity and sick leave [15]. De Bortoli et al. [5] recently found no association between healthy dietary habits and reduced sick leave. Similarly, the relationship between alcohol consumption and sick leave is uncertain, with some studies reporting no increased odds [15,17], while others found no significant association between drinking attitudes and sick leave [29]. These varying results may be due to differences in job types [1,5,15,28]. Ultimately, sick leave is a multifactorial issue influenced not only by lifestyle factors but also by job characteristics [1,30,31] and the overall health status of the working population [3].

In Serbia, 16.4% of residents reported being on sick leave in 2019 [32]. Moreover, over half of the population was either overweight or obese, with 36.0% who were pre-obese and 20.0% who were obese [33]. Daily fruit consumption was reported at 40.0%, while 50.0% consumed vegetables daily, with higher rates observed in Belgrade and among individuals having higher education and better socioeconomic status [32]. Only 11% of adults met the recommended 150 min of weekly aerobic physical activity [32]. Tobacco consumption is a significant concern, with 32.0% of individuals aged 15 and older smoking, more common among men [32]. Alcohol consumption is also notable, with around 50% of the population drinking alcohol, and 3.0% consuming it daily, primarily, men [32]. Despite the recognized link between lifestyle factors and sick leave, comprehensive studies examining the interaction of these factors within the Serbian working population are notably lacking. Previous studies in Serbia have primarily emphasized individual factors including smoking and obesity but have not investigated their combined effects across sexes and occupational groups [32,33]. This gap underscores the need for further investigation to identify at-risk groups and develop targeted interventions [3,5] aimed at mitigating sick leave and improving workforce health in Serbia. Additionally, as underscored by Østby et al. [6], the observed sex differences in sick leave remain largely unexplained, emphasizing the importance of exploring these disparities in greater detail. To the best of our knowledge, there is no specific study on the association between modifiable lifestyle factors and sick leave in Serbia, despite the widespread prevalence of these factors within the Serbian population. The significant burden of obesity, smoking, physical inactivity, and unhealthy dietary habits among the Serbian working population has yet to be thoroughly investigated concerning their association with sick leave.

This study aims to investigate the associations between body mass index, dietary habits (fruit and vegetable intake), physical activity, smoking, and alcohol consumption and sick leave in Serbia, addressing this critical knowledge gap. Furthermore, it will explore whether these associations differ between men and women, contributing to the development of targeted interventions to reduce sick leave and improve workforce health outcomes in Serbia.

## 2. Materials and Methods

### 2.1. Study Design

This study involves a secondary analysis of data from the Serbian National Health Survey of the Republic of Serbia, conducted between October and December 2019. The survey was conducted by the Republic Bureau of Statistics, the Institute of Public Health of Serbia “Dr. Milan Jovanovic Batut”, and the Ministry of Health of the Republic of Serbia. The European Health Survey-third wave (EHIS-wave 3) methodology and resources were employed in the study [34]. Due to the COVID-19 pandemic, the release of the findings was delayed, with results becoming publicly available in 2021.

The study utilized a cross-sectional design with a representative sample of Serbian residents, excluding participants in Kosovo due to unavailable data. A systematic sampling procedure was employed, assigning sampling weights to each participant to ensure the representativeness of the sample relative to the population. These sampling weights were employed in the initial data analysis. The application of sampling weights allowed for the results to be adjusted for the uneven representation of certain demographic groups in the sample, contributing to a more accurate estimation of the relationships between the studied variables. This approach enhanced the generalizability of the findings, increasing the relevance of the study results to the broader population, especially for participants that may have been underrepresented in the sample. This procedure is especially crucial in situations where different demographic groups have varying probabilities of inclusion in the sample, ensuring that no group is disproportionately represented in the final analysis. Consequently, the findings of the study more accurately reflected the actual distribution of characteristics within the target population. The sample was stratified by geographical regions, including Belgrade Region, Vojvodina Region, Šumadija and Western Serbia, Southern Serbia, and Eastern Serbia. The study aimed to include 6000 households, projected to cover around 15,000 individuals aged 15 and older. In total, 5114 households were selected, with 13,178 responses from individuals aged 15 and over, resulting in a response rate of 97.0%. Of the individuals who agreed to participate, 11,790 completed the self-completion form.

For this study, the final sample consisted of 4652 working population, defined as aged 18 to 63 for women and 18 to 65 for men. This age discrepancy reflects the legal retirement ages in Serbia, where women are eligible for retirement at 63 and men at 65 years, following national regulations. Our study focused on participants residing in private homes, excluding those in collective homes or institutions. We excluded individuals outside these age ranges, as well as those with incomplete surveys. This sampling approach ensured that the analysis was representative of the working population within these specified age parameters.

### 2.2. Ethical and Legal Aspects

The study complied with international ethical standards, including the Declaration of Helsinki, and adhered to Serbian legislation. Participants were yielded with a written document explaining the purpose of the study, the scope of their rights, and contact details, including a phone number, for any inquiries or possible complaints. Informed consent was obtained from all participants through written signatures before data collection began. To ensure participants’ privacy and confidentiality, no personally identifiable information was collected, in compliance with the General Data Protection Regulation (GDPR), and Law on Official Statistics. Identifiable data were replaced with codes to maintain anonymity. The anonymized data were securely stored, and reported in aggregate form, in line with the methodology of the European Health Interview Survey—Wave 3 (EHIS—Wave 3) [34]. Permission for the use of secondary data was granted by the Institute of Public Health of Serbia “Dr. Milan Jovanović Batut”, and the database was transferred to the University of Kragujevac, Faculty of Medical Sciences, for further studies.

### 2.3. Survey Instrument

The survey instruments for this study were based on the EHIS Wave 3 questionnaire [34], which adheres to internationally recognized standards and was adapted for the Serbian context. The EHIS Wave 3 questionnaire [34] was selected because it offers comprehensive data on health-related behaviors and socio-economic factors, which are particularly relevant for studying the Serbian population’s lifestyle. Data collection employed three types of questionnaires: a household questionnaire, an adult questionnaire for participants aged 15 and older, and self-completion questionnaires for the same age group. The household questionnaire collected socio-economic information about all members of the household. The adult questionnaire, administered in person, gathered detailed information from participants aged 15 and older. Additionally, self-completion questionnaires allowed participants to yield personal data independently.

For this study, demographic variables (sex, age, region, and marital status), socioeconomic factors (education level and wealth index), and lifestyle factors (body mass index, fruit and vegetable consumption, aerobic physical activity, smoking status, and alcohol consumption) were analyzed. Participants’ sex was self-reported, and the data were analyzed for sex-based differences in sick leave patterns. Age was categorized into five groups: 18–25, 26–35, 36–45, 46–55, and 56–65. Education levels were classified as college/university, secondary, and primary school. Regions of Serbia were divided into Belgrade (Capital), Northern, Central and Western, and Southern and Eastern Serbia. Marital status included categories including married, single, divorced, and widowed. The Wealth Index categorizes households into five quintiles based on their economic status: poorest (Q1), poorer (Q2), middle (Q3), richer (Q4), and richest (Q5). These quintiles, each representing an equal portion of the population, are used to analyze socio-economic disparities among study participants. Occupations were classified according to ISCO08 [35] and EHIS Wave 3 questionnaire recommendation for data dissemination [34] into four groups: managers, professionals, technicians, and associate professionals; clerical support workers, service, and sales workers; skilled manual workers; and elementary occupations. Job physical effort was categorized into heavy (heavy labor or physically demanding work), moderate (tasks involving moderate effort, including walking), and light (mostly sitting or standing). Self-rated health was assessed as very good/good, fair, or bad/very bad.

Participants were categorized according to their body mass index (BMI) using the following classifications: underweight (BMI < 18.5 kg/m^2^), normal weight (BMI between 18.5 and 24.9 kg/m^2^), pre-obesity (BMI between 25 and 29.9 kg/m^2^), and obesity (BMI ≥ 30 kg/m^2^). These categories are based on the World Health Organization’s guidelines [36]. Fruit and vegetable consumption was categorized as daily, 4 to 6 times per week, 1 to 3 times per week, or never/occasionally. Compliance with WHO recommendations for health-enhancing physical activity (HEPA) [37] was assessed by summing the weekly minutes spent cycling and engaging in leisure time sports, fitness, or recreational activities (aerobic physical activity). Based on guidelines recommending 150 min of moderate-intensity activity per week, participants were categorized as high (≥150 min/week), moderate (11–149 min/week), or low (0–10 min/week) in their aerobic physical activity levels. Smoking status was assessed with the question: “Do you smoke tobacco products”? Participants could choose from: “Yes, daily”, “Yes, occasionally”, “Not anymore”, or “I have never smoked.” Responses were classified into never smoker, former smoker, and current smoker. The AUDIT-C evaluated alcohol consumption by calculating daily intake based on participants’ reported consumption [34]. According to Serbian guidelines, low-risk drinking was defined as ≤13 g of pure alcohol per day for women and ≤26 g per day for men [38]. The weekly intake was then calculated by averaging the total volume of alcohol consumed over the past 12 months, measured in grams of pure alcohol per week. Based on this, alcohol consumption was categorized into four groups: lifetime abstainers, former drinkers, non-weekly drinkers, and low-risk drinkers (women: 0–91 g per week, men: 0–182 g per week). Consumption above these thresholds was classified as risky drinking (women: >91 g per week, men: >182 g per week).

The dependent variable is sick leave, assessed based on participants’ reports of missing work due to health reasons in the previous 12 months. Responses were categorized into three groups: no sick leave, short-term sick leave (up to 30 days), and long-term sick leave (over 30 days), reflecting Serbian norms, where the National Health Insurance Fund yields financial assistance to employers following 30 consecutive days of sick leave [39].

### 2.4. Statistical Methods

Chi-square tests (χ^2^) assessed associations between categorical variables, with significance set at *p* < 0.05. Logistic regression models evaluated the associations of the independent variables with sick leave, with results presented as odds ratios (ORs) and 95% confidence intervals (CIs). For the regression analyses, both types of sick leave (short-term and long-term) were combined to provide a comprehensive assessment of overall sick leave patterns. Multivariate models were selected based on theoretical relevance and practical importance, with multicollinearity addressed using variance inflation factors (VIF), with all values below the critical threshold of 5. Model fit was confirmed by the Hosmer–Lemeshow test (*p* > 0.05) and residual analyses showed no significant outliers. Model fit was assessed using Nagelkerke R^2^, and accuracy was confirmed by ROC curve analysis. Cross-validation was carried out using a 10-fold method, ensuring model stability across both urban and rural sub-populations. Outliers and influential data points were checked using Cook’s distance and other metrics, with adjustments made where necessary to maintain model validity. All analyses were conducted with IBM SPSS Statistics, Version 20.0 (IBM Corp., Armonk, NY, USA).

## 3. Results

The study included 4652 participants, with an average age of 42.68 years, categorized by sex. Chi-square tests reveal statistically significant differences across several variables. Women were the most represented in the 36–45 age group (30.8%) (χ^2^ = 17.604, *p* = 0.001). In terms of education, the highest proportion of women had completed college or university (34.8%) compared to men (24.6%), while the majority of men had completed middle school (68.0% vs. 58.1% for women) (χ^2^ = 59.007, *p* < 0.001). Regionally, both sexes were most concentrated in Central and Western Serbia, with men having a higher prevalence (32.9%) compared to women (29.4%) (χ^2^ = 11.964, *p* = 0.008). Marital status data indicate that a higher percentage of women were married (73.1%) compared to men (68.0%) (χ^2^ = 145.876, *p* < 0.001). Regarding the wealth index, women were more represented in the richer class (31.2%) compared to men (27.9%) (χ^2^ = 14.735, *p* = 0.005). Occupational data indicate that men were predominantly engaged in skilled manual work (42.1%), while women were the most represented in managerial or professional roles (38.4%) (χ^2^ = 241.120, *p* < 0.001). In terms of job physical effort, the highest percentage of women were in jobs requiring light or no physical effort (49.6%), whereas men were the most involved in jobs requiring moderate physical effort (43.0%) (χ^2^ = 246.003, *p* < 0.001). Finally, self-rated health data show that a higher percentage of men reported very good or good health (84.5%) compared to women (80.2%) (χ^2^ = 14.611, *p* = 0.001) (Table 1).

As shown in Table 1, there are significant sociodemographic differences between the sexes. Women are more prevalent in the 36–45 age group, have higher education levels, and are more likely to be married and classified as the richest. In contrast, men are the most represented in skilled manual occupations and report good self-rated health.

In total, in the 12 months before the study, 15.8% of participants had sick leave due to personal health issues, with a higher rate among women (18.2%) compared to men (13.9%). Women also had higher rates of both short-term (13.9%) and long-term (3.4%) sick leave compared to men (10.6% and 2.2%, respectively). Age is significantly associated with sick leave, with men aged 56–65 years having the highest proportion of long-term sick leave (46.4%) (χ^2^ = 44.649, *p* < 0.001). Education levels also show a significant relationship with sick leave, with men holding a middle-school education exhibiting the highest prevalence of both short-term (68.0%) and long-term (64.3%) sick leave (χ^2^ = 15.872, *p* = 0.003). Regional disparities were evident, with men from Vojvodina showing the highest prevalence of short-term sick leave (32.4%), while participants from Central and Western Serbia exhibited the highest prevalence of long-term sick leave (35.7%) (χ^2^ = 13.322, *p* = 0.038). Marital status and wealth class did not show statistically significant associations with sick leave, although married men (73.2%) and participants from the richest class (35.7%) had a slightly higher prevalence of long-term sick leave. Occupational categories did not show significant differences; however, skilled manual workers had the highest prevalence of long-term sick leave (44.6%). Job physical effort was marginally non-significant (*p* = 0.057). Finally, self-rated health was strongly associated with sick leave (χ^2^ = 167.279, *p* < 0.001), with men reporting bad or very bad health showing a 17.9% prevalence of long-term sick leave. Men with very good or good self-rated health had significantly higher short-term (68.3%) and long-term (48.2%) sick leave. 57.0 58.9 (Table 2).

As shown in Table 2, men aged 36–45 had the highest short-term sick leave rate at 26.8%, while participants aged 56–65 reported a significant rate of 46.4%. Regional disparities were noted, with the highest rates of short-term sick leave in Vojvodina (35.7%) and long-term sick leave in Central and Western Serbia (35.7%). Additionally, participants with a middle-school education and those who rated their health as good most frequently reported both short-term and long-term sick leave.

Among women, age is significantly associated with sick leave, with women aged 46–55 years showing the highest proportion of short-term sick leave (35.8%) and those aged 36–45 years having the highest proportion of long-term sick leave (31.9%) (χ^2^ = 25.890, *p* = 0.001). Education levels did not show a statistically significant relationship with sick leave, though women with higher education (24.6%) had a slightly higher prevalence of long-term sick leave compared to participants with primary-school education (11.6%) (χ^2^ = 5.126, *p* = 0.275). Regional disparities were evident, with women from Central and Western Serbia showing the highest prevalence of short-term sick leave (35.5%), while women living in Belgrade (the capital) had the highest prevalence of long-term sick leave (34.8%) (χ^2^ = 24.143, *p* = 0.000). Marital status also showed notable distributions in sick leave prevalence, with married women reporting the highest rates of both short-term (71.6%) and long-term (84.1%) sick leave (χ^2^ = 18.935, *p* = 0.004). The wealth index demonstrated a significant relationship, with women from the richest class having the highest prevalence of both short- (37.9%) and long-term (34.8%) sick leave (χ^2^ = 17.746, *p* = 0.023). Occupational categories did not show significant differences. Job physical effort was marginally significant, with women reporting light physical effort having the highest prevalence of short-term sick leave (49.6%) and participants with moderate physical effort showing the highest prevalence of long-term sick leave (44.9%) (χ^2^ = 10.319, *p* = 0.035). Finally, self-rated health showed significant differences in the distribution of sick leave (χ^2^ = 141.699, *p* < 0.001). Women reporting bad or very bad health had a 20.3% prevalence of long-term sick leave, while participants reporting very good or good health had a higher prevalence of both short-term (67.0%) and long-term (44.9%) sick leave (Table 3).

As shown in Table 3, women aged 36–45 had a significantly higher rate of long-term sick leave (31.9%) compared to men (17.9%), indicating workplace disparities associated with health conditions prevalent in this age group. Similar to men, women also displayed regional disparities, with participants rating their health as good experiencing the highest levels of both short-term and long-term sick leave. In contrast to men, married women reported higher rates of both types of sick leave (84.1% compared to 71.6%). Furthermore, women in the richest wealth index class exhibited the highest rates of short-term (37.9%) and long-term (34.8%) sick leave.

Table 4 presents the prevalence of sick leave among men based on lifestyle factors. Among BMI categories, the highest prevalence of both short-term (46.6%) and long-term (43.8%) sick leave was found in participants classified as pre-obese (χ^2^ = 1.375, *p* = 0.967), though these differences were not statistically significant. For fruit consumption, the highest prevalence of short-term sick leave was observed among men consuming fruit four to six times a week (38.3%), while the highest prevalence of long-term sick leave was seen in participants consuming fruit at least once a day (37.7%) (χ^2^ = 9.432, *p* = 0.151), although these differences were not statistically significant. In contrast, men who consumed vegetables at least once a day exhibited the highest prevalence of both short-term (45.2%) and long-term (58.9%) sick leave (χ^2^ = 13.805, *p* = 0.032). Leisure-time aerobic physical activity levels did not show significant differences in sick leave prevalence (χ^2^ = 6.501, *p* = 0.165). Smoking status revealed that never-smokers had the highest prevalence of short-term sick leave (43.8%), while current smokers had the highest prevalence of long-term sick leave (38.1%) (χ^2^ = 12.797, *p* = 0.012). Finally, alcohol consumption patterns indicated that non-weekly drinkers had the highest prevalence of both short-term (35.9%) and long-term (40.5%) sick leave (χ^2^ = 22.491, *p* = 0.004).

Table 5 presents the prevalence of sick leave among women based on lifestyle factors. Among BMI categories, the highest prevalence of both short-term (55.3%) and long-term (38.7%) sick leave was observed in women classified as normal weight (χ^2^ = 17.300, *p* = 0.008). For fruit consumption, the highest prevalence of both short-term (46.5%) and long-term (56.5%) sick leave was found among women consuming fruit at least once a day (χ^2^ = 14.154, *p* = 0.028). Regarding vegetable consumption, women who consumed vegetables at least once a day had the highest prevalence of both short-term (60.6%) and long-term (76.8%) sick leave (χ^2^ = 18.851, *p* = 0.004). Leisure-time aerobic physical activity levels showed that women with low levels of physical activity had the highest prevalence of both short-term (86.4%) and long-term (85.5%) sick leave (χ^2^ = 10.452, *p* = 0.033). Smoking status revealed that never-smokers had the highest prevalence of short-term sick leave (46.2%), while current smokers had the highest prevalence of long-term sick leave (55.4%) (χ^2^ = 18.746, *p* = 0.001). Finally, alcohol consumption patterns indicated that non-weekly female drinkers had the highest prevalence of short-term sick leave (43.9%), while lifetime abstainers had the highest prevalence of long-term sick leave (41.3%). However, the overall differences in alcohol consumption patterns were not statistically significant (χ^2^ = 11.451, *p* = 0.177).

Table 6 presents the association between various lifestyle factors and sick leave among men. When analyzing men, the BMI shows a significant relationship with sick leave across all models. Participants with obesity exhibit more than three times the odds of sick leave compared to participants classified as underweight, with an odds ratio (OR) of 3.007 in Model 1 (CI: 1.607–6.119). Although this association diminishes in Model 4 (OR = 2.613), it remains statistically significant, indicating that obesity continues to be a substantial predictor of sick leave even after adjusting for socio-demographic, occupational, and health factors. Overweight participants also show a significant relationship with sick leave, with OR values ranging from 2.607 to 2.802, necessitating increased odds of sick leave compared to underweight individuals. Normal weight (BMI = 18.5–24.9) has a similar, though slightly lesser, association with sick leave compared to overweight and obesity, with OR values between 2.333 and 2.452, underscoring the significant role of body weight in determining sick leave odds.

Fruit and vegetable consumption has a distinct association with sick leave. Consuming fruit four to six times a week is significantly associated with an increased likelihood of sick leave among men (OR = 1.711 in Model 4). Consuming fruit one to three times a week (OR = 1.819 in Model 1, OR = 1.808 in Model 4) and never/occasionally (OR = 1.713 in Model 1, OR = 2.188 in Model 4) necessitates that regular fruit consumption may act as a protective factor. Similar findings are shown in vegetable consumption. Men who rarely or never consume vegetables exhibit higher odds of sick leave, with OR values ranging from 1.501 to 2.828.

Low leisure-time aerobic physical activity is associated with increased chances of sick leave compared to participants with high physical activity. Odds ratios (OR) for low physical activity range from 1.801 to 1.651, indicating that insufficient physical activity may contribute to higher odds of health problems leading to sick leave among men. Moderate leisure-time aerobic physical activity is associated with an increased likelihood of sick leave (OR = 1.631 in Model 2, OR = 1.601 in Model 4), suggesting that moderate levels of physical activity may not be sufficient to significantly decrease the odds of sick leave among men.

Current male smokers have significantly higher odds of experiencing sick leave compared to non-smokers, with an odds ratio (OR) of 2.016 in Model 1, decreasing slightly to 1.915 in Model 4. This indicates that smoking is a significant predictor of sick leave among men across all models, even after accounting for additional sociodemographic, work-related, and health-related factors. Former male smokers also show increased odds of sick leave (OR = 1.909 in Model 1, OR = 1.713 in Model 4), indicating that even after quitting smoking, the lingering effects of past smoking habits may continue to influence the likelihood of sick leave.

Risky alcohol consumption has the strongest association with sick leave among men, with odds ratios (OR) ranging from 4.004 in Model 2 to 4.111 in Model 4. These values indicate that men engaging in risky drinking have significantly higher odds of sick leave compared to abstainers. Low-risk drinking among men also shows a substantial association with sick leave, with OR values between 3.510 and 3.770, suggesting that moderate alcohol consumption can also increase the odds of health issues leading to sick leave. Non-weekly drinking exhibits significantly higher odds of sick leave, with OR values from 2.512 in Model 1 to 2.818 in Model 4, suggesting that even non-weekly drinking can increase sick leave odds. Former alcohol consumers also exhibit elevated odds for sick leave, with OR values from 3.117 in Model 1 to 3.327 in Model 4, reflecting the lasting health consequences of previous excessive alcohol consumption (Table 6).

Table 7 presents the association between various lifestyle factors and sick leave among women. In women, obesity has a positive but statistically non-significant association with sick leave across all models. In Model 1, the odds ratio (OR) is 2.215 (CI: 0.989–4.967), decreasing to 1.575 in Model 4 (CI: 0.687–3.611) as additional factors are included. Although these values are above 1, the broad confidence intervals indicate that the results are not statistically significant, suggesting that while obesity may increase sick leave odds, additional studies are required to elucidate this association. Overweight shows a similar pattern with OR values between 1.097 and 1.323, but these are also not statistically significant. This implies that women with overweight have slightly higher odds of sick leave compared to underweight individuals, although this association is not substantial. Normal weight demonstrates the least association with sick leave, with OR values ranging from 1.084 to 1.215, indicating that normal weight does not significantly alter the odds of sick leave compared to underweight women.

In women, the consumption of fruit four to six times a week shows an unexpected association. In Model 1, the odds ratio (OR) is 0.915 (CI: 0.639–2.292), and in Models 2, 3, and 4, the values remain below 1, suggesting that regular fruit consumption may reduce the odds of sick leave. However, the wide confidence intervals indicate that these results are not statistically significant. Conversely, occasional or rare fruit consumption (one to three times a week or never/rarely) has OR values above 1, but the confidence intervals are too broad to be statistically significant. These findings indicate that additional studies are required to better understand the association between fruit consumption and its relation to sick leave.

In this analysis, vegetable consumption does not show a significant association with sick leave among women. The odds ratios (OR) for women consuming vegetables less than once a day are below 1 across all models, but the wide confidence intervals suggest that the association may not be strong or is difficult to detect with the available data. This indicates that vegetable consumption may not have a substantial association with sick leave or that the association is not easily observable in this dataset.

Women with low or moderate physical activity levels have increased odds of sick leave compared to participants with high levels of physical activity. The odds ratios (OR) for low physical activity range from 1.551 in Model 1 to 1.361 in Model 4, with similar OR values for moderate physical activity. Although these values are greater than 1, the wide confidence intervals suggest that the results are not statistically significant.

Current female smokers exhibit significantly higher odds of sick leave compared to non-smokers, with odds ratios (OR) ranging from 1.811 in Model 1 to 1.809 in Model 4, indicating consistently high odds. These results are statistically significant, underscoring the need for smoking cessation interventions to reduce sick leave. Former smokers also show increased odds of sick leave, with OR values between 1.803 and 1.967, suggesting that smoking has long-term negative associations with health that persist even after quitting.

Risky alcohol consumption has the strongest association with sick leave among women, with odds ratios (OR) ranging from 2.771 in Model 1 to 3.116 in Model 4. These results suggest that women who engage in risky drinking have significantly increased odds of sick leave compared to participants who abstain from alcohol. However, the wide confidence intervals indicate the need for further studies to confirm these findings. Low-risk alcohol consumers and former drinkers also show slightly elevated odds of sick leave compared to non-drinkers, with OR values just above 1. Although these findings suggest increased odds, the lack of statistical significance underscores the necessity for additional studies (Table 7).

## 4. Discussion

This study aimed to analyze the modifiable lifestyle factors associated with sick leave among Serbia’s working population, with a particular emphasis on sex differences and their association with sick leave. The study included 4652 participants, with an average age of 42.68 years, categorized by sex. The results of the study show significant differences in sociodemographic variables. Women were more represented in the 36–45 age group and had higher educational attainment, with 34.8% completing college or university compared to 24.6% of men. Men were predominantly engaged in skilled manual work, while women were more often in managerial roles. Regional concentration was highest in Central and Western Serbia for both sexes. Additionally, a higher percentage of men reported very good or good health (84.5% vs. 80.2% for women).

### 4.1. Sex Differences

Our study reveals that 15.8% of participants reported sick leave due to personal health issues in the past year, with a higher prevalence among women compared to men. Serbia’s rates are between those of Spain (8.8% for men and 11.9% for women) and the United Kingdom (7.9% for men and 12.3% for women) [1]. Germany reports the highest rates (20.4% for men and 22.9% for women), while Turkey and Romania have decreased rates (Turkey: 2.1% for men and 0.9% for women; Romania: 0.7% for men and 0.4% for women) [1]. The findings reveal significant associations between age, education level, and self-rated health and sick leave. For men, the highest rates of long-term sick leave were observed in participants aged 56–65, with significant regional disparities, especially in Vojvodina and Central and Western Serbia. Men with middle-school education experienced the highest overall sick leave rates. Poor self-rated health was strongly associated with long-term sick leave, indicating a need for targeted interventions for older men, participants with lower education levels, and participants with poor health. Our study found that women had significantly higher rates of both short-term (13.9%) and long-term (3.4%) sick leave compared to men (10.6% and 2.2%, respectively). This disparity may be attributed to a greater prevalence of chronic illnesses, including musculoskeletal disorders, among women [1,3,14]. For women, significant factors associated with sick leave included age, marital status, wealth index, and self-rated health. Women aged 46–55 had the highest rates of short-term sick leave, while participants aged 36–45 had the highest rates of long-term sick leave. Regional disparities were also evident, with Central and Western Serbia showing the highest short-term sick leave rates and Belgrade having the highest long-term sick leave prevalence. Additionally, married women and participants from higher wealth classes had more frequent sick leave, and light physical job effort was associated with higher short-term sick leave, whereas moderate effort was associated with long-term sick leave. Women with poor self-rated health had the highest rates of long-term sick leave. Our findings align with those of previous studies [1,23,40] that emphasize the association between socio-demographic factors, self-rated health, and sick leave. For instance, Lidwall [41] noted sex segregation in occupational sick leave patterns. Contrary to the study suggesting that women in lower wealth classes have higher sick leave rates [31], our results show that women from higher wealth classes reported more frequent sick leave. This discrepancy calls for further investigation into the association between wealth index classes and sick leave. Additionally, while Mensah et al. [30] found that men reported poorer self-rated health compared to women across 30 European countries, our study found that females more frequently rated their health as less than very good. These inconsistencies suggest potential variations in health perception and reporting. Our results underscore the need for sex-specific health interventions in Serbia, including better access to healthcare, flexible workplace policies, and enhanced social support for women. Targeted strategies are essential for addressing the needs of older male workers, participants with lower education levels, and participants with poor health. A comprehensive approach that considers both sex-specific and broader demographic factors is crucial for developing effective interventions to reduce absenteeism and improve health outcomes.

### 4.2. Impact of Obesity

The analysis of lifestyle factors revealed interesting patterns in the prevalence of sick leave, though many of these differences were not statistically significant. The regression analysis reveals notable associations between BMI and sick leave for both sexes, though patterns differ. For men, obesity is significantly associated with sick leave, with participants classified as obese having over three times the odds of taking sick leave in an unadjusted model compared to underweight individuals. This association remains statistically significant even after adjusting for sociodemographic, occupational, and health factors. Pre-obese male participants also have increased odds of sick leave. In contrast, the association between obesity and sick leave for women is positive but not statistically significant, indicating that while there are increased odds of sick leave for obese women, the evidence is not strong enough to be conclusive. Pre-obese women also show slightly increased odds of sick leave compared to underweight women, but this finding is not substantial. Overall, while obesity is a clear predictor of sick leave among men, the relationship for women is less pronounced and requires further investigation to elucidate underlying factors. Several factors may contribute to the observed differences in sick leave between sexes. Men may exhibit distinct health-seeking behaviors, often failing to recognize or report obesity-related symptoms compared to women. Additionally, they are typically employed in more physically demanding jobs, which can exacerbate health conditions associated with obesity, leading to increased sick leave. Societal expectations regarding masculinity and the stigma of obesity may further influence men’s responses to health issues. Our findings align with many previous studies indicating a strong link between obesity and sick leave [4,7,8,42], although some studies suggest varying results due to differences in job types [15,28]. Such discrepancies may arise from differences in the occupations of the study populations.

### 4.3. Combined Risk Factors

The results of the study reveal distinct patterns regarding fruit and vegetable consumption and its association with sick leave, with notable differences between sexes. The results of the study reveal distinct patterns regarding fruit and vegetable consumption and their association with sick leave, with notable differences between male and female participants. Among men, consuming fruit never or occasionally or one to three times a week is associated with increased odds of sick leave, suggesting that regular fruit consumption may serve as a protective factor. Similar trends are observed with vegetable consumption, underscoring the importance of a balanced diet in maintaining health and mitigating the odds of sick leave. These findings align with a previous study by Canerva et al. [12], which emphasizes the role of fruit and vegetable intake in promoting overall health. For women, the relationship between fruit and vegetable consumption and sick leave appears complex. Women who consume fruit and vegetables daily report higher prevalence rates of both short-term and long-term sick leave, suggesting that participants with healthier dietary habits may be more proactive in managing underlying health conditions that require time off work. Despite this, the data do not demonstrate a strong protective association between daily fruit and vegetable consumption and the mitigation of sick leave. Notably, while regular fruit consumption (four to six times a week) seems to indicate a potential reduction in sick leave odds in regression analysis, this finding is not statistically significant. Similarly, vegetable consumption does not show a significant association with sick leave, with results indicating that any potential link may be weak or difficult to detect. These findings underscore the need for further studies to fully understand the influence of fruit and vegetable consumption on sick leave among women. Overall, while regular fruit and vegetable consumption seems to help reduce sick leave among men, the relationship for women is more complex and may be influenced by other factors that need further exploration. This variation in findings is not surprising, as some studies, including those by Fitzgerald et al. [9], have demonstrated a positive association between a healthy diet and the mitigation of sick leave, while others, including De Bortoli et al. [5], found no significant association. These differences may be attributed to several factors. Women may be more likely to seek medical advice and manage health issues, resulting in higher sick leave despite healthier dietary habits. Additionally, societal expectations around health and diet may lead women to report their health conditions more openly, impacting their sick leave patterns. Moreover, hormonal and biological differences between sexes may influence how diet compromises health and sick leave.

The results of the study show that, among men, low or moderate levels of leisure-time aerobic physical activity are associated with higher chances of sick leave compared to high physical activity levels. Specifically, men with low and moderate levels of leisure-time aerobic physical activity show a higher prevalence of sick leave, suggesting that even moderate levels of physical activity may not be sufficient to significantly decrease the odds of sick leave among men. Similarly, for women, those with low and moderate levels of leisure-time aerobic physical activity also show a higher prevalence of sick leave. This finding is consistent with those of several studies indicating that insufficient physical activity is associated with increased sick leave [4,11,16]. Although our results indicate that women with low or moderate physical activity levels have higher odds of sick leave compared to participants with high physical activity levels, these results were not statistically significant. This suggests that further studies with more precise measurements or larger sample sizes are required to elucidate these associations. Overall, these findings underscore the importance of regular physical activity in potentially mitigating sick leave. They underscore the role of an active lifestyle in maintaining overall health and suggest that promoting higher levels of physical activity could help decrease sick leave rates. Differences in physical activity levels between sexes may be attributed to occupational demands, with men often engaged in more physically demanding jobs. Women’s physical activity may vary due to caregiving responsibilities and societal expectations, which can impact their health and sick leave patterns. Psychological and emotional factors may also play a role in these differences. Insufficient leisure-time aerobic activity could increase the risk of health issues, leading to higher rates of sick leave.

Our study reveals that smoking status is a significant determinant of sick leave, with notable differences among current smokers, former smokers, and never-smokers. Both current smokers and former smokers exhibit a significantly higher prevalence of long-term sick leave, consistent with the established health risks associated with smoking. This suggests that smoking cessation programs could be particularly effective in mitigating long-term sick leave among both men and women. For men, current smokers have notably higher odds of sick leave compared to never-smokers, reinforcing smoking as a major OR factor for sick leave even when adjusted for sociodemographic, work-related, and health-related factors. Former smokers also show notably higher odds of sick leave, indicating that the negative association between health and smoking may persist over time, though their odds of sick leave are decreased compared to current smokers. Similarly, among women, current smokers have significantly higher odds of sick leave, with the data underscoring smoking as a major factor associated with increased odds of long-term sick leave. Former female smokers demonstrate even more elevated odds of sick leave, suggesting that smoking’s adverse health effects can continue to influence health even after quitting. These findings underscore the importance of targeted smoking cessation interventions to mitigate sick leave. Our results align with previous studies showing a strong association between smoking and increased sick leave [3,4], emphasizing the need for effective smoking cessation strategies to improve overall health outcomes and reduce absenteeism. In terms of smoking behaviors, men may be less likely to acknowledge smoking-related health issues, potentially contributing to differences in health outcomes between sexes. Social and cultural factors may also influence smoking perceptions, underscoring the importance of tailoring smoking cessation programs to address these nuances for both sexes.

For men, results from regression analysis revealed that risky drinking is a particularly significant predictor, with men engaging in risky alcohol consumption showing the highest odds of sick leave. Low-risk and non-weekly drinking among men also shows a substantial association with sick leave, indicating that any level of alcohol consumption can increase the odds of health leading to sick leave. Former drinkers also display elevated odds of sick leave, underscoring the lasting health association with previous alcohol consumption. For women, the pattern is different. Risky drinking is associated with increased odds of sick leave, but the results are less conclusive, with broad confidence intervals suggesting a need for further study. Low-risk drinkers and former alcohol consumers show only a slight elevation in odds of sick leave compared to non-drinkers, and these findings are not statistically significant, indicating that alcohol’s association with sick leave among women may be less pronounced or more complex. Some authors [15,17] have found that alcohol consumption does not elevate the odds of sick leave; others have found no drinking attitudes differences in regard to the influence on sick leave [29]; however, many more studies [20,40,43] confirm the association between alcohol consumption and increased odds of sick leave in both men and women. The observed sex differences may be attributed to variations in drinking patterns and health-seeking behaviors, with men potentially experiencing more immediate health consequences from alcohol use. Cultural perceptions of alcohol consumption may also influence how both sexes experience its effects on health and subsequent sick leave.

This analysis underscores the significant association between lifestyle factors including BMI, dietary habits, smoking, alcohol consumption, and sick leave among men in Serbia. The findings underscore the importance of targeted interventions to reduce sick leave and improve health outcomes in male workers. In contrast, the analysis of sick leave among women presents a more complex picture, with mixed associations between lifestyle factors and sick leave. While factors including smoking and risky alcohol consumption are associated with increased sick leave, others, including BMI, fruit and vegetable consumption, and physical activity, show less consistent patterns. This suggests that the association between lifestyle factors and sick leave among women may be more nuanced and require further investigation to fully understand. The absence of a significant association between alcohol consumption and sick leave among women is particularly surprising, suggesting that the relationship may be more relevant for short-term rather than long-term sick leave. In addition to the factors examined in our study, some research has identified associations with certain socio-psychological factors, including psychosocial work environment [16] and stress [18], further highlighting the complexity of this issue.

Understanding modifiable lifestyle factors is essential for developing effective interventions to reduce sick leave and improve productivity. To address the factors influencing sick leave, several targeted strategies could be employed. Firstly, implementing comprehensive workplace wellness programs that emphasize weight management can be beneficial, offering nutritional guidance and promoting physical activity. Secondly, establishing smoking cessation initiatives can support employees who wish to quit smoking, mitigating related health issues. Additionally, improving access to healthier food options and providing nutrition education within the workplace could enhance fruit and vegetable consumption. For alcohol-related concerns, increasing awareness about its health effects and providing resources for individuals struggling with alcohol consumption could be effective. Regular health screenings can further assist in the early detection and prevention of health problems. These approaches aim to improve overall employee health, reduce sick leave, and boost productivity.

### 4.4. Limitations

This study has several limitations that must be acknowledged. First, the reliance on self-reported data may introduce bias due to inaccurate reporting of health behaviors, including potential underreporting of alcohol consumption influenced by social desirability bias. Recall bias may also compromise the accuracy of self-reported lifestyle data. Furthermore, the use of specific cut-points for health behaviors and sick leave may not align with participants employed in other studies, thus complicating comparisons. The 30-day sick leave threshold limits the generalizability of findings to studies utilizing different cut-points and to other populations outside Serbia. Missing data and non-participation may also compromise the overall findings. The higher prevalence of self-completion questionnaires due to the sensitivity of questions on smoking and alcohol consumption could potentially influence results. The cross-sectional design of the study precludes the establishment of temporal relationships between variables, meaning the findings cannot imply causality. This design constrains our understanding of how lifestyle behaviors influence sick leave over time.

The study did not account for specific physical or mental illnesses that could significantly influence both lifestyle behaviors and sick leave and could potentially influence results. These unmeasured factors may confound the results and should be considered when interpreting the findings. Demographic characteristics, including being married, possessing a middle-school education, and belonging to higher occupational classes, may lead to conservative findings, skewing results. The study emphasized behavioral factors compromising sick leave, while socio-psychological factors, including psychosocial work environment, stress, and workplace support, were not considered. Future studies should incorporate these variables for a comprehensive understanding of sick leave causes.

The inability to conduct separate regression analyses for short-term and long-term sick leave was due to the imbalance in the sample. The number of participants with long-term sick leave was significantly lower compared to short-term sick leave, which could lead to unreliable and statistically unfavorable results. Therefore, both types of sick leave were analyzed together to achieve stability in the conclusions. Future studies with larger samples could enable separate analyses for short-term and long-term sick leave. Additionally, the non-inclusion of sampling weights in the regression analysis, despite the application of a systematic sampling procedure, may pose a limitation in terms of generalizing the findings to a broader population. Although participants were selected based on homogeneous characteristics, mitigating the need for weighting corrections, future studies could benefit from including sampling weights in the regression analysis to enhance the generalizability of results, particularly in larger and more diverse samples.

The study’s strengths include a representative sample of Serbia’s working population, achieved through a rigorous stratified two-stage sampling method. Following the European Health Interview Survey methodology study ensures international comparability, and using a national census framework supports the reliability of findings while including reserve households to help address non-response bias.

## 5. Conclusions

This study elucidates the significant association between lifestyle factors with sick leave within the Serbian working population, with a particular emphasis on sex differences. For male participants, key factors including obesity, dietary habits, smoking, and alcohol consumption consistently predict the likelihood of sick leave. The implementation of effective interventions that target weight management, dietary improvements, smoking cessation, and the reduction of risky alcohol consumption may significantly decrease sick leave rates. These measures have the potential to enhance individual health and alleviate burdens on healthcare systems and employers.

In contrast, the findings for female participants indicate that smoking and risky alcohol consumption are the most substantial predictors of sick leave. This underscores the urgent need for public health initiatives aimed at mitigating these risks. While BMI and physical activity are associated with sick leave, their role in this study was not statistically significant, necessitating the influence of other factors and the need for further investigation. The role of fruit and vegetable consumption remains ambiguous, with wide confidence intervals necessitating additional studies to elucidate its association with sick leave. Further studies should investigate the combined effects of physical activity and job types on sick leave, as well as cultural factors influencing dietary reporting in the Serbian context.

This study has yielded significant insights into the impact of modifiable lifestyle factors on sick leave, analyzing the sociodemographic characteristics of the targeted sample while focusing on sex differences. The combined analysis of short-term and long-term sick leave offers a comprehensive understanding of these factors’ overall impact. Although sampling weights were not incorporated into the regression analysis due to sample homogeneity, the results nonetheless illuminate the dynamics of sick leave within the working population. Future studies should consider integrating sampling weights in regression analyses to enhance the generalizability of findings to a broader population.

Further analyses that separately investigate factors influencing short-term and long-term sick leave could deepen the understanding of absenteeism and its causes. Additionally, incorporating socio-psychological factors including stress, job satisfaction, work-life balance, and workplace support could further illuminate the complexity of sick leave across different work environments. By integrating physical, behavioral, and socio-psychological factors, future studies could develop more targeted interventions to reduce sick leave and enhance the health of the working population, providing a broader perspective on the factors influencing sick leave across various professions and work environments.

Overall, this study emphasizes the necessity for tailored interventions and ongoing studies to better understand and address the diverse modifiable lifestyle factors related to sick leave across both sexes.

## Figures and Tables

**Table 1:** Sociodemographic description of participants.

Variables	Sex				Pearson Chi-Square/df/*p* *
	Male		Female		
	N	%	N	%	
Age					
18–25	206	7.9	109	5.3	17.604/4/0.001
26–35	584	22.5	450	21.9	
36–45	721	27.7	633	30.8	
46–55	668	25.7	556	27.1	
56–65	420	16.2	305	14.9	
Education					
College/University	638	24.6	714	34.8	59.007/2/0.000
Middle school	1767	68.0	1193	58.1	
Primary school	192	7.4	146	7.1	
Region of Serbia					
Belgrade (Capital)	645	24.8	589	28.7	11.964/3/0.008
Vojvodina	625	24.0	508	24.7	
Central and Western Serbia	855	32.9	603	29.4	
Southern and Eastern Serbia	474	18.2	353	17.2	
Marital status					
Married	1762	68.0	1495	73.1	145.876/3/0.000
Single	696	26.9	316	15.5	
Divorced	121	4.7	167	8.2	
Widowed	13	0.5	67	3.3	
Wealth index (class)					
Richest	726	27.9	640	31.2	14.735/4/0.005
Richer	640	24.6	531	25.9	
Middle	540	20.8	418	20.4	
Poor	413	15.9	297	14.5	
Poorest	280	10.8	167	8.1	
Occupations					
Managers, professionals, and technicians	710	27.6	779	38.4	241.120/3/0.000
Clerical support, service, and sales workers	621	24.1	623	30.7	
Skilled manual workers	1086	42.1	421	20.7	
Elementary occupations	160	6.2	207	10.2	
Job physical effort					
Heavy	517	21.2	104	5.3	246.003/2/0.000
Moderate	1050	43.0	889	45.1	
None/light	877	35.9	979	49.6	
Self-rated health					
Very good/good	2067	84.5	1583	80.2	14.611/2/0.001
Fair	322	13.2	328	16.6	
Bad/very bad	56	2.3	63	3.2	

* Statistical significance was set at *p* < 0.05.

**Table 2:** Prevalence of sick leave by sociodemographic characteristics—male.

Variables	Sick Leave						
	No		Short-Term		Long-Term		Pearson Chi-Square/df/*p* *
	N	%	N	%	N	%	
Age							
18–25	186	8.3	15	5.5	2	3.6	44.649/8/0.000
26–35	505	22.6	66	24.3	5	8.9	
36–45	629	28.1	73	26.8	10	17.9	
46–55	578	25.8	69	25.4	13	23.2	
56–65	340	15.2	49	18.0	26	46.4	
Education							
College/University	568	25.4	53	19.5	14	25.0	15.872/4/0.003
Middle school	1520	68.0	185	68.0	36	64.3	
Primary school	148	6.6	34	12.5	6	10.7	
Region of Serbia							
Belgrade (Capital)	554	24.8	67	24.6	15	26.8	13.322/6/0.038
Vojvodina	521	23.3	88	32.4	10	17.9	
Central and Western Serbia	752	33.6	75	27.6	20	35.7	
Southern and Eastern Serbia	411	18.4	42	15.4	11	19.6	
Marital status							
Married	1498	67.1	196	72.3	41	73.2	7.975/6/0.240
Single	618	27.7	62	22.9	10	17.9	
Divorced	105	4.7	11	4.1	5	8.9	
Widowed	11	0.5	2	0.7	0	0.0	
Wealth index (class)							
Richest	623	27.8	75	27.6	20	35.7	12.648/8/0.125
Richer	561	25.1	61	22.4	14	25.0	
Middle	448	20.0	70	25.7	11	19.6	
Poor	374	16.7	31	11.4	6	10.7	
Poorest	232	10.4	35	12.9	5	8.9	
Occupations							
Managers, professionals, and technicians	626	28.2	66	24.4	15	26.8	5.579/6/0.472
Clerical support, service, and sales workers	538	24.2	63	23.2	12	21.4	
Skilled manual workers	926	41.7	118	43.5	25	44.6	
Elementary occupations	129	5.8	24	8.9	4	7.1	
Job physical effort							
Heavy	432	20.6	72	26.5	8	14.3	9.171/4/0.057
Moderate	906	43.1	116	42.6	22	39.3	
None/light	763	36.3	84	30.9	26	46.4	
Self-rated health							
Very good/good	1848	87.9	185	68.3	27	48.2	167.279/4/0.000
Fair	226	10.8	71	26.2	19	33.9	
Bad/very bad	28	1.3	15	5.5	10	17.9	

* Statistical significance was set at *p* < 0.05.

**Table 3:** Prevalence of sick leave by sociodemographic characteristics—female.

Variables	Sick Leave						
	No		Short-Term		Long-Term		Pearson Chi-Square/df/*p* *
	N	%	N	%	N	%	
Age							
18–25	99	5.9	9	3.2	0	0.0	25.890/8/0.001
26–35	382	22.8	40	14.2	18	26.1	
36–45	511	30.5	93	33.0	22	31.9	
46–55	431	25.7	101	35.8	19	27.5	
56–65	254	15.1	39	13.8	10	14.5	
Education							
College/University	581	34.6	104	36.9	17	24.6	5.126/4/0.275
Middle school	981	58.5	158	56.0	44	63.8	
Primary school	115	6.9	20	7.1	8	11.6	
Region of Serbia							
Belgrade (Capital)	456	27.2	99	35.1	24	34.8	24.143/6/0.000
Vojvodina	430	25.6	54	19.1	18	26.1	
Central and Western Serbia	483	28.8	100	35.5	17	24.6	
Southern and Eastern Serbia	308	18.4	29	10.3	10	14.5	
Marital status							
Married	1214	72.7	202	71.6	58	84.1	18.935/6/0.004
Single	278	16.7	33	11.7	3	4.3	
Divorced	125	7.5	35	12.4	6	8.7	
Widowed	52	3.1	12	4.3	2	2.9	
Wealth index (class)							
Richest	501	29.9	107	37.9	24	34.8	17.746/8/0.023
Richer	444	26.5	67	23.8	15	21.7	
Middle	342	20.4	60	21.3	9	13.0	
Poor	251	15.0	31	11.0	10	14.5	
Poorest	139	8.3	17	6.0	11	15.9	
Occupations							
Managers, professionals, and technicians	624	37.7	112	39.9	30	43.5	7.314/6/0.293
Clerical support, service, and sales workers	521	31.5	76	27.0	20	29.0	
Skilled manual workers	352	21.3	55	19.6	11	15.9	
Elementary occupations	159	9.6	38	13.5	8	11.6	
Job physical effort							
Heavy	76	4.7	18	6.4	9	13.0	10.319/4/0.035
Moderate	729	45.5	124	44.0	31	44.9	
None/light	796	49.7	140	49.6	29	42.0	
Self-rated health							
Very good/good	1348	84.1	189	67.0	31	44.9	141.699/4/0.000
Fair	227	14.2	74	26.2	24	34.8	
Bad/very bad	28	1.7	19	6.7	14	20.3	

* Statistical significance was set at *p* < 0.05.

**Table 4:** Prevalence of sick leave according to lifestyle factors—male.

Variables	Sick Leave						
	No		Short-Term		Long-Term		Pearson Chi-Square/df/*p* *
	N	%	N	%	N	%	
Body mass index							
Underweight	16	0.9	1	0.4	0	0.0	1.375/6/0.967
Normal weight	496	27.8	68	28.8	15	31.3	
Pre-obese	841	47.2	110	46.6	21	43.8	
Obese	428	24.0	57	24.2	12	25.0	
Fruit consumption							
At least once a day	766	37.6	81	33.3	20	37.7	9.432/6/0.151
4 to 6 times a week	158	29.3	15	38.3	13	24.5	
1 to 3 times a week	597	25.3	93	22.2	15	28.3	
Never/occasionally	516	7.8	54	6.2	5	9.4	
Vegetable consumption							
At least once a day	999	47.5	123	45.2	33	58.9	13.805/6/0.032
4 to 6 times a week	649	30.9	71	26.1	16	28.6	
1 to 3 times a week	393	18.7	64	23.5	5	8.9	
Never/occasionally	60	2.9	14	5.1	2	3.6	
Leisure-time aerobic physical activity							
High	191	9.1	23	8.8	2	3.8	6.501/4/0.165
Moderate	374	17.8	50	19.2	4	7.7	
Low	1542	73.2	188	72.0	46	88.5	
Smoking status							
Never smoker	788	49.2	91	43.8	14	33.3	12.797/4/0.012
Former smoker	194	12.1	30	14.4	12	28.6	
Current smoker	620	38.7	87	41.8	16	38.1	
Alcohol consumption							
Lifetime abstainers	366	25.2	29	15.8	6	16.2	22.491/8/0.004
Former drinking	131	9.0	19	10.3	3	8.1	
Non-weekly drinking	599	41.3	66	35.9	15	40.5	
Low-risk drinking	295	20.3	60	32.6	12	32.4	
Risky drinking	61	4.2	10	5.4	1	2.7	

* Statistical significance was set at *p* < 0.05.

**Table 5:** Prevalence of sick leave according to lifestyle factors—female.

Variables	Sick Leave						
	No		Short-Term		Long-Term		Pearson Chi-Square/df/*p* *
	N	%	N	%	N	%	
Body mass index							
Underweight	39	2.9	5	2.1	1	1.6	17.300/6/0.008
Normal weight	784	57.6	130	55.3	24	38.7	
Pre-obese	381	28.0	62	26.4	21	33.9	
Obese	158	11.6	38	16.2	16	25.8	
Fruit consumption							
At least once a day	689	43.0	131	46.5	39	56.5	14.154/6/0.028
4 to 6 times a week	832	52.0	130	46.1	25	36.2	
1 to 3 times a week	69	4.3	18	6.4	3	4.3	
Never/occasionally	11	0.7	3	1.1	2	2.9	
Vegetable consumption							
At least once a day	869	54.3	171	60.6	53	76.8	18.851/6/0.004
4 to 6 times a week	496	31.0	71	25.2	12	17.4	
1 to 3 times a week	204	12.8	32	11.3	4	5.8	
Never/occasionally	31	1.9	8	2.8	0	0.0	
Leisure-time aerobic physical activity							
High	144	9.0	19	7.2	3	4.3	10.452/4/0.033
Moderate	191	11.9	17	6.4	7	10.1	
Low	1265	79.1	229	86.4	59	85.5	
Smoking status							
Never smoker	689	56.0	102	46.2	21	37.5	18.746/4/0.001
Former smoker	116	9.4	32	14.5	4	7.1	
Current smoker	425	34.6	87	39.4	31	55.4	
Alcohol consumption							
Lifetime abstainers	500	46.8	79	42.2	19	41.3	11.451/8/0.177
Former drinking	93	8.7	12	6.4	8	17.4	
Non-weekly drinking	412	38.6	82	43.9	15	32.6	
Low-risk drinking	59	5.5	13	7.0	3	6.5	
Risky drinking	4	0.4	1	0.5	1	2.2	

* Statistical significance was set at *p* < 0.05.

**Table 6:** Unadjusted and adjusted odds ratio (OR) with a 95% confidence interval (CI) for the association between lifestyle factors and overall sick leave—male.

Variables		Model 1 OR (95% CI)	Model 2 OR (95% CI)	Model 3 OR (95% CI)	Model 4 OR (95% CI)
Body mass index	Underweight	1	1	1	1
	Normal weight	2.501 (1.201–5.111) *	2.333 (1.115–4.754) *	2.411 (1.158–4.800) *	2.452 (1.202–5.103) *
	Pre-obese	2.802 (1.405–5.640) *	2.607 (1.318–5.211) *	2.730 (1.351–5.425 *	2.662 (1.363–5.287) *
	Obese	3.007 (1.607–6.119) *	2.819 (1.568–5.659) *	2.850 (1.504–5.704) *	2.613 (1.414–5.200) *
Fruit consumption	At least once a day	1		1	1
	4 to 6 times a week	1.506 (0.912–2.705)	1.562 (0.901–2.805)	1.607 (0.951–2.904)	1.711 (1.001–3.119) *
	1 to 3 times a week	1.819 (1.119–3.052) *	1.852 (1.153–3.060) *	1.850 (1.107–3.088) *	1.808 (1.115–3.010) *
	Never/occasionally	1.713 (1.139–2.576) *	2.050 (1.357–3.272) *	2.103 (1.488–3.334) *	2.188 (1.367–3.339) *
Vegetable consumption	At least once a day	1	1	1	1
	4 to 6 times a week	0.870 (0.570–1.328)	0.910 (0.592–1.399)	0.910 (0.591–1.401)	0.921 (0.593–1.431)
	1 to 3 times a week	0.949 (0.570–1.580)	0.947 (0.563–1.591)	0.983 (0.583–1.657)	0.986 (0.579–1.679)
	Never/occasionally	1.501 (0.901–2.702)	2.744 (1.081–6.965) *	2.749 (1.079–7.000) *	2.828 (1.086–7.367) *
Leisure-time aerobic physical activity	High	1	1	1	1
	Moderate	1.514 (0.910–2.702)	1.631 (1.095–2.801) *	1.552 (0.902–2.700)	1.601 (1.007–2.814) *
	Low	1.801 (1.011–3.201) *	1.751 (0.977–3.179)	1.704 (0.901–3.052)	1.651 (0.844–2.944)
Smoking status	Never smoker	1	1	1	1
	Former smoker	1.909 (1.169–3.406) *	1.861 (1.037–3.308) *	1.809 (1.007–3.313) *	1.713 (1.116–3.165) *
	Current smoker	2.016 (1.207–3.404) *	1.910 (1.520–3.304) *	1.858 (1.109–3.305) *	1.915 (1.161–3.376) *
Alcohol consumption	Lifetime abstainers	1	1	1	1
	Former drinking	3.117 (1.837–5.620) *	3.129 (1.834–5.811) *	3.241 (1.915–6.005) *	3.327 (1.910–6.201) *
	Non-weekly drinking	2.512 (1.602–4.211) *	2.613 (1.650–4.370) *	2.659 (1.652–4.488) *	2.818 (1.712–4.608) *
	Low-risk drinking	3.510 (2.101–6.108) *	3.593 (2.271–6.271) *	3.613 (2.249–6.332) *	3.770 (2.262–6.412) *
	Risky drinking	4.153 (2.301–6.410) **	4.004 (2.958–6.405) **	4.001 (2.942–6.273) **	4.111 (2.829–6.884) **

Statistical significance: *—*p* < 0.05; **—*p* < 0.01. Model 1: unadjusted odds ratio for lifestyle-related factors with sick leave. Model 2 adjusted the odds ratio for Model 1 with the addition of sociodemographic factors with sick leave. Model 3 adjusted the odds ratio for Model 2 with the addition of work-related factors with sick leave. Model 4 adjusted the odds ratio for Model 3 with the addition of health-related factors with sick leave.

**Table 7:** Unadjusted and adjusted odds ratio (OR) with a 95% confidence interval (CI) for the association between lifestyle factors and overall sick leave—female.

Variables		Model 1 OR (95% CI)	Model 2 OR (95% CI)	Model 3 OR (95% CI)	Model 4 OR (95% CI)
Body mass index	Underweight	1	1	1	1
	Normal weight	1.215 (0.556–2.487)	1.112 (0.446–2.324)	1.084 (0.451–2.287)	1.125 (0.489–2.414)
	Pre-obese	1.323 (0.609–2.873)	1.119 (0.493–2.538)	1.097 (0.479–2.518)	1.154 (0.505–2.642)
	Obese	2.215 (0.989–4.967)	1.759 (0.767–3.732)	1.701 (0.748–3.738)	1.575 (0.687–3.611)
Fruit consumption	At least once a day	1		1	1
	4 to 6 times a week	0.915 (0.639–2.292)	0.972 (0.664–1.472)	0.998 (0.679–1.436)	0.968 (0.644–1.346)
	1 to 3 times a week	1.245 (0.590–2.593)	1.456 (0.678–3.218)	1.523 (0.694–3.331)	1.258 (0.567–2.763)
	Never/occasionally	2.315 (0.468–11.465)	2.594 (0.517–12.915)	2.738 (0.558–13.527)	2.703 (0.535–14.872)
Vegetable consumption	At least once a day	1	1	1	1
	4 to 6 times a week	0.840 (0.534–1.321)	0.867 (0.545–1.380)	0.822 (0.513–1.316)	0.920 (0.566–1.493)
	1 to 3 times a week	0.639 (0.344–1.185)	0.668 (0.354–1.262)	0.655 (0.345–1.244)	0.716 (0.371–1.381)
	Never/occasionally	1.055 (0.303–3.677)	1.369 (0.380–4.929)	1.472 80.397–5.456)	1.280 (0.323–5.076)
Leisure-time aerobic physical activity	High	1	1	1	1
	Moderate	1.512 (0.872–2.621)	1.479 (0.856–2.578)	1.461 (0.847–2.583)	1.187 (0.702–2.011)
	Low	1.551 (0.891–2.701)	1.482 (0.859–2.612)	1.432 (0.820–2.511)	1.361 (0.775–2.387)
Smoking status	Never smoker	1	1	1	1
	Former smoker	1.950 (1.151–3.310) *	1.803 (1.045–3.019) *	1.917 (1.106–3.302) *	1.967 (1.167–3.384) *
	Current smoker	1.811 (1.261–2.601) *	1.879 (1.341–2.635) *	1.812 (1.302–2.602) *	1.809 (1.221–2.651) *
Alcohol consumption	Lifetime abstainers	1	1	1	1
	Former drinking	1.611 (0.721–3.521)	1.591 (0.707–3.501)	1.581 (0.702–3.481)	1.681 (0.747–3.781)
	Non-weekly drinking	1.512 (0.872–2.621)	1.523 (0.879–2.652)	1.552 (0.878–2.651)	1.575 (0.912–2.741)
	Low-risk drinking	1.462 (0.689–3.101)	1.459 (0.688–3.095)	1.491 (0.703–3.211)	1.561 (0.735–3.411)
	Risky drinking	2.771 (0.721–15.482) *	2.931 (0.815–16.431) *	3.051 (0.851–17.501) *	3.116 (0.872–18.112) *

Statistical significance: *—*p* < 0.05; Model 1: unadjusted odds ratio for lifestyle-related factors with sick leave. Model 2 adjusted the odds ratio for Model 1 with the addition of sociodemographic factors with sick leave. Model 3 adjusted the odds ratio for Model 2 with the addition of work-related factors with sick leave. Model 4 adjusted the odds ratio for Model 3 with the addition of health-related factors with sick leave.

## Data Availability

Due to privacy and ethical restrictions, the data used in this study are not available for public access. The rights to the data are held by the Institute of Public Health of Serbia, “Dr. Milan Jovanović Batut”. The database was officially transferred to the University of Kragujevac, Faculty of Medical Sciences, to conduct further research.
